# The utility of chest computed tomography (CT) and RT-PCR screening of asymptomatic patients for SARS-CoV-2 prior to semiurgent or urgent hospital procedures

**DOI:** 10.1017/ice.2020.331

**Published:** 2020-07-16

**Authors:** Aditya S. Shah, Lara A. Walkoff, Ronald S. Kuzo, Matthew R. Callstrom, Michael J. Brown, Michael L. Kendrick, Bradly J. Narr, Elie Berbari

**Affiliations:** 1 Division of Infectious Disease, Department of Medicine, Mayo Clinic, Rochester, Minnesota; 2 Department of Radiology, Mayo Clinic, Rochester, Minnesota; 3 Department of Anesthesiology, Mayo Clinic, Rochester, Minnesota; 4 Department of Surgery, Mayo Clinic, Rochester, Minnesota

## Abstract

**Objective:**

Presently, evidence guiding clinicians on the optimal approach to safely screen patients for coronavirus disease 2019 (COVID-19) to a nonemergent hospital procedure is scarce. In this report, we describe our experience in screening for SARS-CoV-2 prior to semiurgent and urgent hospital procedures.

**Design:**

Retrospective case series.

**Setting:**

A single tertiary-care medical center.

**Participants:**

Our study cohort included patients ≥18 years of age who had semiurgent or urgent hospital procedures or surgeries.

**Methods:**

Overall, 625 patients were screened for SARS-CoV-2 using a combination of phone questionnaire (7 days prior to the anticipated procedure), RT-PCR and chest computed tomography (CT) between March 1, 2020, and April 30, 2020.

**Results:**

Of the 625 patients, 520 scans (83.2%) were interpreted as normal; 1 (0.16%) had typical features of COVID-19; 18 scans (2.88%) had indeterminate features of COVID-19; and 86 (13.76%) had atypical features of COVID-19. In total, 640 RT-PCRs were performed, with 1 positive result (0.15%) in a patient with a CT scan that yielded an atypical finding. Of the 18 patients with chest CTs categorized as indeterminate, 5 underwent repeat negative RT-PCR nasopharyngeal swab 1 week after their initial swab. Also, 1 patient with a chest CT categorized as typical had a follow-up repeat negative RT-PCR, indicating that the chest CT was likely a false positive. After surgery, none of the patients developed signs or symptoms suspicious of COVID-19 that would indicate the need for a repeated RT-PCR or CT scan.

**Conclusion:**

In our experience, chest CT scanning did not prove provide valuable information in detecting asymptomatic cases of SARS-CoV-2 (COVID-19) in our low-prevalence population.

Coronavirus disease 2019 (COVID-19), caused by severe acute respiratory syndrome coronavirus 2 (SARS-CoV-2), was first described in Wuhan, China, in December 2019 and was subsequently declared a global pandemic by the WHO on March 11, 2020.^1-3^ Unprecedented measures have been implemented worldwide in an effort to flatten the curve of this pandemic. A key measure, early in the pandemic, relied on postponing and cancelling elective hospital procedures to limit the spread of COVID-19 and to preserve much needed healthcare resources. This delay of elective procedures, if protracted, may adversely affect the clinical care and outcome of patients without COVID-19. Patients with undiagnosed or asymptomatic COVID-19 pose a risk to both health care workers and other patients. Therefore, measures aimed at detecting patients with asymptomatic COVID-19 infection before a planned procedure or surgery should be implemented. Although more data are needed regarding the sensitivity of reverse-transcriptase polymerase chain reaction (RT-PCR), the definitive test for COVID-19 infection, reports available currently and reported in the Infectious Disease Society of America (IDSA) guidelines for diagnosis of COVID-19, estimate it to be between 75% and 95%.^4^ The reported sensitivity of chest computed tomography scans (CTs) for patients with COVID-19 pneumonia varies but has been reported to be as high as 98%.^5^ Given the potential for false-negative RT-PCR tests, we sought to determine whether the addition of a second test with high sensitivity, such as chest CT, could enhance the detection of patients with asymptomatic COVID-19. Safely managing patients in need of elective hospital procedures will continue to be relevant during this pandemic and beyond. Here, we describe our experience and the results of implementing this safety project of screening and testing patients for SARS-CoV-2 (COVID-19) prior to semiurgent or urgent hospital procedures using both CT chest imaging and RT-PCR testing.

## Methods

Our institution screened surgical patients preoperatively using a 3-pronged approach: patient phone interview, CT imaging of the chest, and SARS-CoV-2 nasopharyngeal swab testing by RT-PCR. 7 days prior to the anticipated procedure, patients were contacted by phone. Using a standardized questionnaire, they were evaluated for symptoms of fever, cough, shortness of breath, difficulty in breathing, sore throat, diarrhea, chills, or myalgia, and for exposures to COVID-19 infection such as high-risk travel or contact. An affirmative response to any of these questions would result in deferral of the surgical procedure, if feasible. A clinical team would then contact the patient to formulate a treatment plan.

If the phone-screening questionnaire was entirely negative, the patient would undergo a SARS-CoV-2 nasopharyngeal swab PCR 48 hours prior to the elective hospital procedure as well as CT imaging of the chest the day before the procedure. Our initial plan was to swab patients 5 days and 2 days prior to planned procedure; however, this procedure changed to just 2 days (48 hours) prior to the procedure due to limited testing availability and significant logistical difficulties.

In the large majority of cases, chest CT was performed using a low radiation dose protocol without intravenous (IV) contrast material. Exceptions were made in several instances in which concurrent staging for malignancy was requested in addition to screening for SARS-CoV-2, in which case standard-dose chest CT imaging was performed with or without IV contrast. In all cases, the radiologist was provided with 0.75-mm-thick contiguous axial slices reconstructed at 0.5-mm intervals and 3-mm-thick contiguous axial, coronal, and sagittal series. The CT chest study was interpreted as typical, indeterminate, atypical, or normal using the criteria set forth in the publication “Radiological Society of North America Expert Consensus statement on reporting chest CT findings related to COVID-19, endorsed by the Society of Thoracic Radiology, the American College of Radiology, and RSNA” and the corresponding “suggested reporting language” accompanied each category in the impression of the CT report.^6^ Typical CT features were defined as those reported in literature as associated with COVID-19 pneumonia, including peripheral bilateral ground glass opacities (GGOs) or multifocal GGOs of a rounded morphology (with or without consolidation or intralobular lines), or findings of organizing pneumonia. An indeterminate study was defined as the absence of typical features and the presence of “multifocal, diffuse, perihilar, or unilateral GGO with or without consolidation lacking a specific distribution and are non-rounded or non-peripheral” or “few very small GGOs with a non-rounded and non-peripheral distribution.” An atypical appearance was defined as the absence of typical or indeterminate features and having isolated lobar or segmental consolidation without GGOs, discrete small nodules, cavitation, or smooth interlobular septal thickening with a pleural effusion. A normal study was defined as one without features to suggest pneumonia.^6-8^ Comparison to prior chest CTs was made, if available.

If the SARS-CoV-2 PCR was negative and the chest CT was interpreted as either normal or atypical for COVID-19, the patient underwent their planned procedure. In the setting of either a positive SARS-CoV-2 PCR or a chest CT with typical findings for COVID-19, the procedure was deferred. When indeterminate chest CT findings were present and SARS-CoV-2 PCR was negative, management was determined on an individual patient basis.

## Results

In total, 625 patients underwent imaging and RT-PCR testing. Results are summarized in Table 1. Our cohort of 625 patients had chest CTs performed with RT-PCR testing (640 RT-PCR tests). Of the 625 patients, 520 scans (83.2%) were interpreted as normal; 1 (0.16%) had typical features; 18 scans (2.88%) had indeterminate features; and 86 (13.76%) had atypical features of COVID-19. Only 1 of the 625 screening chest CTs was performed on a patient with a positive RT-PCR result. In total, 640 RT-PCRs were performed, with 1 positive result (0.15%). This positive result was in a patient with CT scan interpreted as atypical.

**Table 1.** Summary of Results

Imaging (n = 625)	Age, Mean (Range)	Sex, Female, No. (%)	RT-PCR Positive
Typical (n = 1)	41	1 (100)	0
Indeterminate (n = 18)	60 (31–81)	8 (44)	0
Atypical (n = 86)	63 (30–95)	37 (43)	1
Normal (n = 520)	58 (18–90)	220 (42)	0

Note. RT-PCR, reverse transcriptase polymerase chain reaction.

Of the 18 patients with chest CTs categorized as indeterminate, 5 underwent repeat RT-PCR nasopharyngeal swab 1 week after their initial negative swab; all results remained negative. Also, 1 patient with chest CT categorized as typical had a follow-up repeat RT-PCR which was negative, indicating that the chest CT was likely a false positive.

After surgery, none of the patients developed signs or symptoms suspicious of COVID-19, needing repeat RT-PCR or CT scan.

## Discussion

Our results demonstrate that while feasible, screening chest CT provided little additional value for the detection of SARS-CoV-2 in asymptomatic individuals when performed in conjunction with a screening questionnaire and RT-PCR in our population, where there is a low presence of COVID-19 (~20 cases per 100,000 population).^9^ Several studies examining the utility of chest CT in the diagnosis of COVID-19 pneumonia have recently been performed. Multiple studies have demonstrated the presence of bilateral peripheral GGOs with a lower-lung predominance as one of the typical features of RT-PCR diagnosed COVID-19 pneumonia^10,11^; however, “typical” chest CT findings for COVID-19 can also be seen in other entities including infectious processes, organizing pneumonia, and connective-tissue diseases.^6^ Current studies have demonstrated that chest CT is able to discern pulmonary findings in patients with microbiologically diagnosed COVID-19 pneumonia with a sensitivities up to 90% and specificities up to 96%.^12,13^ Several reports in the literature advocate the use of chest CT as a reliable alternative to RT-PCR.^14^ Notably, however, these studies were performed on largely symptomatic groups of patients in regions with a high population prevalence of SARS-CoV-2,^12-15^ which contrasts with our entirely asymptomatic cohort living in a low-prevalence region. A recent meta-analysis evaluating the performance of CT and RT-PCR in the diagnosis of COVID-19 showed an overall pooled sensitivity of 94% and specificity of 37% for chest CT with a pooled sensitivity of RT-PCR being 89%; it concluded that in regions with low disease prevalence, the positive predictive value of RT-PCR was ~10 times that of a CT.^16^ Another study revealed that 56% of patients with COVID-19 had negative chest CTs within the first 2 days of symptom onset.^17^

Among asymptomatic patients on the *Diamond Princess* cruise ship who tested positive for SARS-CoV-2 by RT-PCR, CTs scan were negative for pulmonary opacities in 46% of cases,^18^ although the prevalence of disease was relatively high in this cohort (~26%). With regard to RT-PCR, initial reports indicate a sensitivity of between 75% and 95% in patients with symptoms of the disease.^4^

In many cases, the patient’s underlying condition may have predisposed them to having lung findings, appears similar to COVID-19 pneumonia. The patient with CT findings interpreted as being typical for COVID-19 had a history of systemic lupus erythematosus, which may have produced the lung parenchymal abnormalities. Many of the patients had a history of malignancy that had been treated with chemotherapy and radiation before the planned surgical procedure. The high prevalence of nonspecific lung findings, which were likely due to other causes, made it difficult to completely exclude COVID pneumonia and resulted in false-positive CT interpretations. Thus, CTs might perform better when used in an otherwise healthy population.

One of the strengths of this study is the large number of patients involved in the screening process. Our hope is that our findings help guide other healthcare systems as they begin to resume routine clinical operations.

The low prevalence of SARS-CoV-2 (COVID-19) in our local community could be a potential limitation of this study because the positive predictive value of our approach might be hampered by low community prevalence. However, our findings would be applicable to other regions with a similar community prevalence of SARS-CoV-2 (COVID-19). Our findings have the potential to save patients from unnecessary testing, expense, and potential delays in elective hospital procedures.

In this evolving global pandemic of COVID-19, safe resumption of surgical and interventional procedures is critical for patient care and health care, both of which have been drastically adversely impacted. With the goal of resuming semiurgent and urgent procedural care at our institution, we took a conservative approach to minimize the risks to both patients and providers, using questionnaires and RT-PCR in combination with screening chest CT. In our experience, chest CT scanning did not prove provide valuable information in detecting asymptomatic cases of COVID-19 in our low-prevalence population. Our findings are in keeping with statements by multiple organizations, including the Center for Disease Control and Prevention (CDC) and the Society of Thoracic Radiology, which do not recommend routine CT for the diagnosis of patients under investigation for COVID-19.
